# Meropenem Disposition in Neonatal and Pediatric Extracorporeal Membrane Oxygenation and Continuous Renal Replacement Therapy

**DOI:** 10.3390/antibiotics13050419

**Published:** 2024-05-03

**Authors:** Pavla Pokorná, Danica Michaličková, Dick Tibboel, Jonas Berner

**Affiliations:** 1 Institute of Pharmacology, First Faculty of Medicine, Charles University and General University Hospital, 128 00 Prague, Czech Republic; 2 Department of Pediatrics and Inherited Metabolic Disorders, First Faculty of Medicine, Charles University and General University Hospital, 128 00 Prague, Czech Republic; 3 Department of Physiology and Pharmacology, Karolinska Institute and Karolinska University Hospital, 171 77 Stockholm, Sweden; 4 Department of Pediatric Surgery, Erasmus Medical Center Sophia Children’s Hospital, 3062 PA Rotterdam, The Netherlands; 5 Pediatric Perioperative Medicine and Intensive Care, Astrid Lindgren Children’s Hospital, Karolinska University Hospital, 171 76 Stockholm, Sweden

**Keywords:** extracorporeal membrane oxygenation, meropenem, continuous renal replacement therapy, Monte Carlo simulations, neonates, children

## Abstract

This study aimed to characterize the impact of extracorporeal membrane oxygenation (ECMO) on the pharmacokinetics (PK) of meropenem in neonates and children and to provide recommendations for meropenem dosing in this specific population of patients. Therapeutic drug monitoring (152 meropenem plasma concentrations) data from 45 patients (38 received ECMO) with a body weight (BW) of 7.88 (3.62–11.97) kg (median (interquartile range)) and postnatal age of 3 (0–465) days were collected. The population PK analysis was performed using NONMEM V7.3.0. Monte Carlo simulations were performed to assess the probability of target achievement (PTA) for 40% of time the free drug remained above the minimum inhibitory concentration (fT > MIC) and 100% fT > MIC. BW was found to be a significant covariate for the volume of distribution (Vd) and clearance (CL). Additionally, continuous renal replacement therapy (CRRT) was associated with a two-fold increase in Vd. In the final model, the CL and Vd for a typical patient with a median BW of 7.88 kg that was off CRRT were 1.09 L/h (RSE = 8%) and 3.98 L (14%), respectively. ECMO did not affect meropenem PK, while superimposed CRRT significantly increased Vd. We concluded that current dosing regimens provide acceptably high PTA for MIC ≤ 4 mg/L for 40% fT > MIC, but individual dose adjustments are needed for 100% fT > MIC.

## 1. Introduction

Extracorporeal membrane oxygenation (ECMO) is a treatment modality used in critically ill neonates and children with severe respiratory and/or circulatory failure [1]. Pharmacotherapy in ECMO is complicated due to maturational changes in pharmacokinetics (PK) and pharmacodynamics (PD), leading to variable drug dispositions and unpredictable treatment outcomes [2]. In general, critical illness and ECMO treatment may lead to PK changes due to an altered volume of distribution (Vd) and/or drug clearance (CL) [3,4]. ECMO-induced PK changes are responsible for the increase in Vd due to dynamically changed drug adsorption/sequestration, compound inactivation and hemodilution, as reported in vitro [5,6]. The degree of drug adsorption in the ECMO circuit is highly variable and depends on the physicochemical properties of the drug, such as molecular size, drug lipophilicity and plasma protein binding, and is determined by the interaction of the drug with different types of ECMO circuits, oxygenators or pumps. Additionally, the duration of ECMO treatment was also found to be a significant PK covariate [7].

Meropenem is a broad-spectrum antimicrobial agent, which is commonly administered in critically ill neonates and children because of its broad antimicrobial spectrum and favorable safety profile [8]. Meropenem exhibits time-dependent antimicrobial activity and can be administered by intermittent bolus infusion, by prolonged infusion (2–4 h) or continuous infusion (24 h). The suggested PD target for susceptible bacteria is 40% of time the free drug remains above the minimum inhibitory concentration (40% fT > MIC). To achieve this, a standard dose (20 to 40 mg/kg infused over 30 min every 8 h) is administered. In case of severe infection, the attainment of 100% fT > MIC has been recommended. To reach this target, continuous infusion (60–120 mg/kg per day) is administered, but only in infants and children [9,10].

Meropenem PK is characterized by an extracellular distribution, both through the hepatic and renal metabolisms. The renal excretion of meropenem is mediated by glomerular filtration and tubular secretion, while almost 70% of given meropenem is recovered intact in the urine within 12 h [11]. Meropenem PK is dependent on maturation in neonates, specifically on the postnatal age (PNA), body weight (BW) and estimated glomerular filtration rate (e-GFR) [12]. The impact of non-maturational factors on the PK of meropenem, such as critical illness or continuous renal replacement therapy (CRRT), are still debated [13,14,15,16,17]. Data on the impact of ECMO and/or covariates (CRRT) on the PK of meropenem are limited [18]. Here, we focused on population PK studies using the non-linear model effect (NLME) approach. Small studies using a population PK approach in pediatric patients (excluding neonates) found no effect of ECMO on meropenem PK [19,20,21]. Additionally, three case reports [22,23,24] and a study not using the NLME approach [25] yielded mixed results. Interestingly, ex vivo studies have found a significant loss of meropenem within an ECMO circuit with an oxygenator in the first 24 h after application of meropenem, although meropenem is a hydrophilic drug [26,27]. Considering the small sample sizes of the published studies, we need more data on meropenem in neonates and children undergoing ECMO to achieve meropenem efficacy and a safety profile [18]. The primary objective of this pilot study was to characterize the PK of meropenem in critically ill neonates and children undergoing ECMO. A secondary objective was to propose optimal meropenem dosing recommendations using Monte Carlo simulations in this population.

## 2. Results

### 2.1. Patient Population and Therapeutic Drug Monitoring

Therapeutic drug monitoring (TDM) data from 45 critically ill neonates and children were available for the analysis (see Figure S1 in Supplementary Materials); 38 underwent the ECMO treatment (oxygenators and ECMO pumps were used for ages 0–18 years: oxygenators—HILITE 2400 and 7000 Quadrox; ECMO pumps—DP3 and Centrimag) and 31 CRRT (either continuous veno-venous hemodialysis (CVVHD) or hemodiafiltration (CVVHDF) treatment modalities), of which 19 (61%) were treated with CRRT for fluid overload (FO) [28] and 12 (39%) for acute kidney injury (AKI), while 3 patients without CRRT had moderate AKI according to Kidney Disease: Improving Global Outcomes (KDIGO) criteria [29,30,31,32,33]. When using continuous renal replacement therapy (CRRT) (Prismaflex, Baxter) in the ECMO treatment, a pre-pump was connected, i.e., drainage and return were on the low-pressure side before the ECMO pump and standard dialysis solutions (Hemosol (dialysate and replacement)) in patients with body weight < 10 kg and mostly used Hemosol (dialysate and substitute) in patients with a body weight of 10–30 kg; only in a few cases Phoxilium was used. The clinical characteristics of the patients are provided in Table 1. There were 152 available plasma concentrations ranging from 0.68 to 75 mg/L, of which 94 were collected during the ECMO treatment, 91 plasma concentrations during CRRT and 61 off CRRT. Details on the number and frequency of meropenem concentrations on/off ECMO and CRRT are given in Table S1 in the Supplementary Materials.

### 2.2. Population Pharmacokinetic Model

Observed meropenem plasma concentrations were most accurately represented by a one-compartment model with log-normally distributed intra-individual variability (IIV) on CL and Vd. The best description of residual variability was achieved with a proportional error model. The incorporation of BW as a continuous covariate in a linear relationship on CL significantly improved the model fit (*p* < 0.001). Likewise, BW was identified as a significant covariate for Vd (*p* < 0.001) and was also included as a continuous covariate in a linear relationship on Vd. Both Vd and CL were normalized to a median BW value of the cohort. Incorporating CRRT as a binary covariate (i.e., on/off) on Vd led to a significant improvement in the model fit (*p* < 0.01). None of the other covariates showed statistical significance after adding this covariate relationship. The final parameter estimates can be found in Table 2. In the final model, the CL and Vd for a typical patient of the median BW of 7.88 kg that was off CRRT were 1.09 L/h (RSE = 8%)) and 3.98 L (14%), respectively. CRRT was found to increase Vd approximately two times.

**Figure 1:**
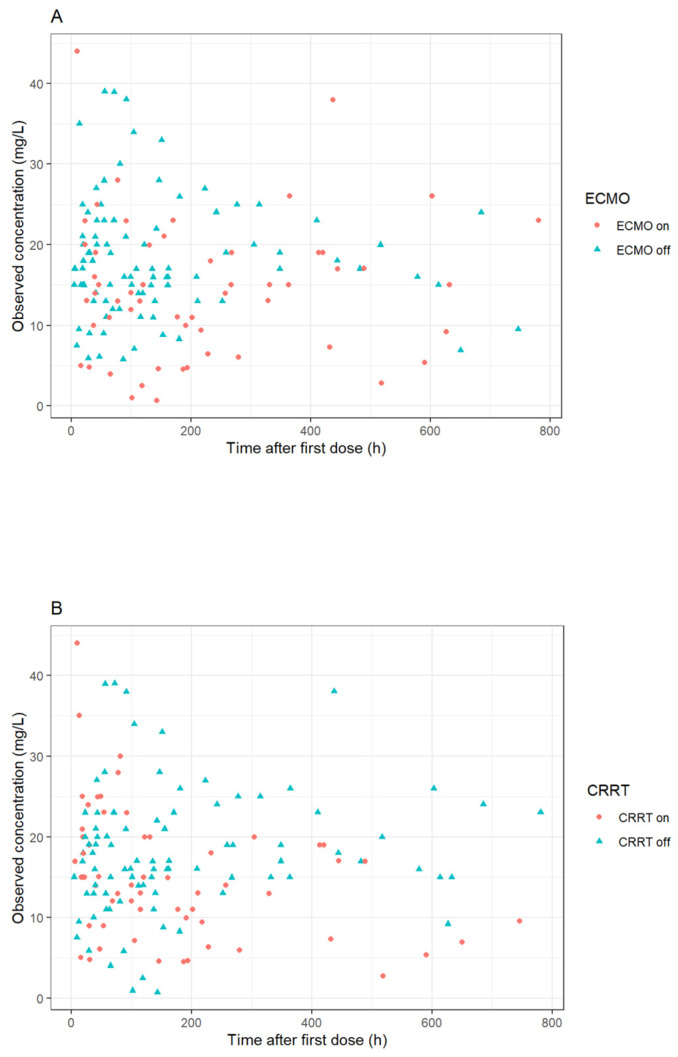
Meropenem concentrations vs. time after last dose stratified according to (**A**) extracorporeal membrane oxygenation (ECMO) and (**B**) continuous renal replacement therapy (CRRT).

**Figure 2:**
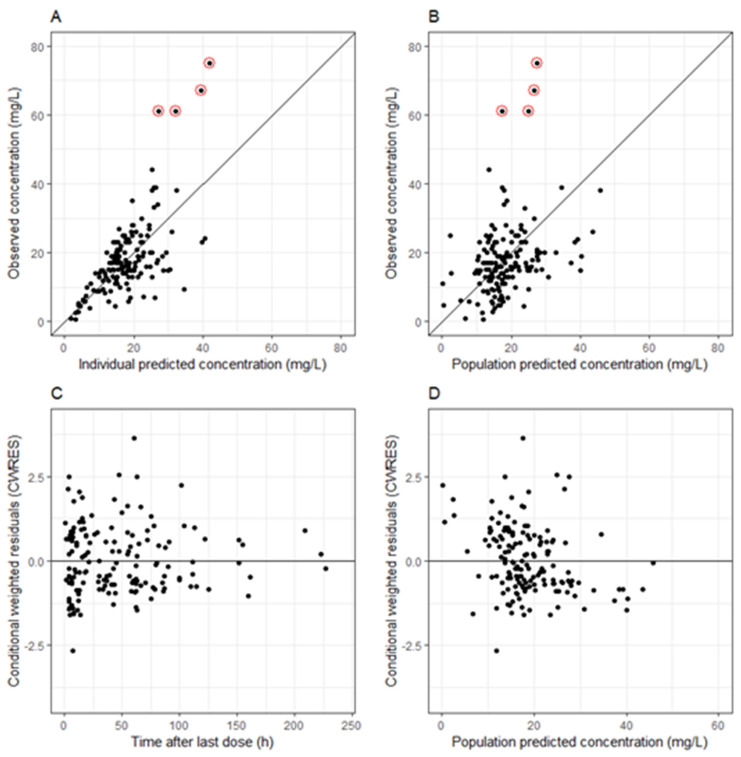
Goodness of fit plots for the final model of meropenem pharmacokinetics in neonatal and pediatric populations. (**A**) Population-predicted meropenem concentration vs. observed meropenem concentration. (**B**) Individual-predicted meropenem concentration vs. observed meropenem concentration. (**C**) Conditional weighted residuals (CWRES) vs. time after dose. (**D**) Conditional weighted residuals (CWRES) vs. population-predicted concentrations.

Apart from θCRRT and IIV for Vd, the precision of the estimated parameter values was satisfactory, with RSE values below 50%. Regardless of the increased uncertainty in the estimated values, we chose to maintain the covariate relationship in the model due to its significant clinical relevance, as advised by clinicians. The basic GOF plots in Figure 1 suggested that the final model accurately represented the data, showing only a minor trend in conditional weighted residuals (CWRES) versus population-predicted concentrations. No bias in basic GOF plots for individual and population concentrations was observed, apart from four outlier concentrations (Figure 2), which were much lower than the observed. These concentrations were measured during meropenem infusion, and we presume that a medical error occurred during the collection of these samples.

Except for IIV for Vd, median parameter values calculated with the bootstrap procedure were within 10% of the values acquired in the final model fit, suggesting that the model results were robust. For IIV for Vd, the bootstrap median deviated 13% from the final parameter estimate. The final parameter value fell comfortably within the 95% bootstrap interval, enhancing the confidence in the estimated value. The distribution of the NPDEs obtained with the model for the dataset (Figure S1 in Supplementary Materials) had a mean of −0.05 (SE = 0.081) and variance of 1.007 (SE = 0.12). Neither of these values were significantly different from the expected values of 0 (*p* = 1) and 1 (*p* = 1), respectively. This suggested that the predictions for the structural model and the data variability were accurate.

### 2.3. Simulations for Meropenem Dose Optimization

PTA for 40% fT > MIC and 100% fT > MIC for BW = 7.88 kg (for a typical individual) are given in Table 3. PTA > 90% was highlighted. PTA obtained for BW of 3.92 kg and 11.97 kg was given in the Supplementary Materials.

## 3. Discussion

This was a retrospective pilot study using a population pharmacokinetics approach to describe the PK of meropenem to achieve PD goals in neonatal and pediatric ECMO settings. Although other studies looked at the PK of meropenem in pediatric ECMO, this study included the highest number of patients, while simultaneously comparing ECMO with non-ECMO subgroups. Our PK analysis showed that ECMO modality did not affect the PK of meropenem in this population, while BW was a significant covariate for the CL and Vd. Additionally, Vd was found to be increased approximately twofold in CRRT patients superimposed on ECMO. Finally, model-based Monte Carlo simulations were performed to provide the optimal dosing of meropenem in this population.

We found a one-compartment model to provide the best description of meropenem distribution. This was in line with several studies with critically ill neonatal and pediatric patients [12,14,16,17,20,34], whereas other studies found a two-compartment model to ensure the best fit [13,15,19,21,35]. Reported values for CL and Vd normalized to BW in studies with critically ill pediatric patients were in the range 0.057–0.63 L/h/kg and 0.46–2.04 L/kg, respectively [13,15,16,17,19,20,21,34,35]. Our BW-normalized values of CL and Vd were 0.138 L/h/kg and 0.51 L/kg, respectively, which was in the reported range.

To date, three small studies using the NMLE approach testing ECMO effects on meropenem PK in pediatric patients have been published. In a study of 25 patients, Wang et al. found that BW and estimated creatinine clearance (e-CRCL) affected CL, whereas BW was found to be covariate for Vd [20]. Moreover, the authors found CRRT and ECMO not to have impact on the PK/PD parameters. Similarly, in a study of nine patients, Zylbersztajn et al. reported that ECMO did not influence PK parameters [21]. BW was also found to be a covariate for Vd. Finally, a study of nine critically ill children receiving CRRT by Tan et al. did not aim primarily to assess the effects of ECMO on meropenem PK, but tested ECMO as a covariate for Vd and CL [19]. The authors did not find the impacts of either ECMO or CRRT on PK to be significant and attained only an allometric relationship with total BW with fixed estimates of 0.75 for CL and 1.0 for Vd in the final model. Moreover, a study using a non-compartmental method approach also did not find any effect of ECMO on meropenem PK [25]. On the other hand, three previously published case reports observed mixed results: Cies et al. reported ECMO to increase meropenem CL [22], Jabareen et al. found an ECMO-induced reduction in meropenem CL [23], while Saito et al. observed decreased CL and increased Vd of meropenem [24]. It is worth mentioning that an ex vivo study found a loss of meropenem within an ECMO circuit with an oxygenator in the first 24 h after the application of meropenem [26,27].

Regarding CRRT’s effect on PK, published data in critically ill neonatal and pediatric patients are debatable. Similarly, the influence of different CRRT modalities (CVVHD or CVVHDF) on the PK of meropenem is only partially understood. Our results corroborated findings from a previous study analyzing meropenem PK in critically ill children, in which CRRT increased Vd by 66% [15]. This could be explained by the fact that patients receiving CRRT are often fluid-overloaded, and this can give rise to an increase in Vd, which is especially pronounced with hydrophilic drugs, such as meropenem [36]. Conversely, in the aforementioned studies by Wang et al. and Tan et al., CRRT influenced neither meropenem Vd nor CL [19,20]. In a recent study, however, Thy et al. found that the CRRT parameter total effluent flow affected CL in critically ill children [16]. Additionally, a study by Rapp et al., found that CRRT had a major role in meropenem’s elimination of pediatric patients with different renal functions [10]. Given these considerations, a large-scale study is required to investigate the impact of CRRT settings on meropenem PK in pediatric and neonatal patients.

CrCL was found to be a significant covariate in several studies [15,17,20], but not in all studies evaluating critically ill neonatal and pediatric patients. We also did not find either creatinine or CrCL to be significantly associated with meropenem CL, and this can likely be explained by the fact that creatinine is not a reliable surrogate marker of kidney function in the first week of life. It is worth noting that a large proportion of patients in this cohort comprised neonates (60%).

It is generally known that the management of AKI depends on the definitions of AKI used. Currently, a modified definition of KDIGO based on the Schwartz equation for GFR estimation is being created (we also used the Schwarz equation in our analysis) [37]. This fact seems to be promising for understanding the diagnosis of AKI in critically ill children and optimizing treatment with meropenem. The AKI assessment in our cohort was performed according to the KDIGO criteria; however, a prospective validation is important at this stage to better understand the effect of CRRT indicated for FO and/or AKI during ECMO on meropenem PK. Our findings indicated that critical illness indeed raised the volume of distribution (Vd) of hydrophilic drugs like meropenem, and this hypothesis was confirmed in both scenarios—when CRRT was utilized for FO and/or AKI.

Although our model was demonstrated to accurately describe data through diagnostic and validation procedures (GOFs, NPDE and the bootstrap method), it is important to note four outlier concentrations (Figure 2). In these instances, the predicted levels were considerably lower than the observed ones. The suspected cause is an error occurring during sample collection, particularly as these concentrations were measured during meropenem infusion. Such incidents are anticipated in real-world studies, where data control is not as stringent as in randomized clinical trials (RCTs) [38]. RCTs operate under highly controlled conditions, whereas our study examined data from routine clinical practice, offering potentially more applicable clinical insights. Other limitations of the study were the sparse data collection at early time points after ECMO onset, as well as the inclusion of children up to 5 years of age. In the future, our goal is to thoroughly examine the concentrations present throughout the entire duration of the ECMO treatment, spanning from its initiation to its conclusion, for patients up to 18 years old.

According to the Monte Carlo simulations (Table 3 and Tables S2 and S3 in the Supplementary Materials), very similar results were obtained for all three BW categories (25th percentile, median and 75th percentile of our cohort, these were 3.62, 7.88 and 11.97 kg, respectively) when comparing the same dosing regimens normalized for BW. The only exception was a high PTA (>90%) obtained also for neonates not receiving CRRT for 100% fT > MIC; as for the other two BW categories, this result was obtained only for patients receiving CRRT. For well-susceptible pathogens to meropenem with MICs below 2 mg/L, for which meropenem monotherapy is recommended, all patients achieved a high PTA for 40% fT > MIC with meropenem administered either 20–40 mg/kg over a 30 min short infusion or a 3 h prolonged infusion three times daily or by continuous infusion with 60 or 120 mg/kg/day, regardless of the therapy with CRRT. In contrast, for susceptible pathogens with MICs ≥ 0.5 mg/L and <2.0 mg/L, the only regimen achieving a high PTA for 100% fT > MIC was a continuous administration of 120 mg/kg/day for all three categories of simulated BW (while receiving CRRT). This result was also achieved for neonates (typically a 3.62 kg patient) not receiving CRRT. For the so-called “grey zone” of pathogens with MICs between 2 and 8 mg/L, where a higher dose of meropenem or combination antibiotic treatment is usually recommended, the simulations showed the following: PTA for 40% fT > MIC was achieved for MIC up to 4 mg/L across all BW categories, treatment modalities and dosing regimens of meropenem except for short infusion of 20–40 mg/kg over 30 min in patients not undergoing CRRT. The no dosing regimen achieved a high PTA for 100% fT > MIC. Our findings were similar to the recommendations provided by Wang et al. [20].

## 4. Materials and Methods

### 4.1. Study Design

A retrospective observational study was conducted in critically ill neonates and children (0–5 years old) treated with meropenem admitted to the Department of Pediatric Perioperative Medicine and Intensive Care and ECMO, Astrid Lindgren Children’s Hospital, Karolinska University Hospital, Stockholm, Sweden, in cooperation with the First Faculty of Medicine, Charles University, Czech Republic, and Data Sharing Consensus. Approval of the study was provided by the ethical committee of the General University Hospital in Prague under RV project 128/22 S-IV EC approval in 2022, and the Protocol Identifying Number K 2021-6676 at Karolinska University Hospital, Stockholm, Sweden (5 August 2021), and ethical committee approval from the Swedish Ethical Review Authority with Protocol Identifying Number Dnr 2021-02390. This study included retrospective patient records from a patient data management system (Take care digital journal system Compu Group, Stockholm, Sweden; Centricity Critical Care Clinisoft, GE Healthcare Europe, Stockholm, Sweden) from Pediatric Perioperative Medicine and Intensive Care, and ECMO Centre Astrid Lindgren Children’s Hospital, Karolinska, University Hospital, Stockholm, Sweden, from 1 January 2018 to 31 December 2020.

The major indications for ECMO in this cohort were pneumonia, acute respiratory distress syndrome, septic shock, respiratory insufficiency, meconium aspiration and congenital diaphragmatic hernia. In PICU patients who did not receive ECMO, the indications were congenital diaphragmatic hernia, macrophage activation syndrome, persistent pulmonary hypertension of the newborn, pneumonia, suspected or proven sepsis and septic shock.

Positive blood cultures were determined as follows: *Pseudomonas aeruginosa*, *Stenotrophomonas maltophilia*, *Escherichia coli* (n = 2), *Staphylococcus aureus*, *Staphylococcus epidermidis*, *Neisseria meningitidis* (MIC = 0.002 mg/L), Coagulase-negative *Staphylococcus* (n = 2), *Hemophilus influenzae* (MIC= 0.25 mg/L) and *Streptococcus bovis*.

### 4.2. Meropenem Dosing

Meropenem was administered as prescribed by the physicians to infants younger than 3 months of age over a 30 min short infusion and over 15–30 min of short infusion in older children intravenously using a gestational (GA) and PNA intermittent dosing approach from 20 to 40 mg/kg 2–4 times a day, according to the local protocols at the time of the study. During the ECMO treatment, meropenem was administered by continuous infusion after a loading dose of 20 mg/kg (maximum 2 g) in the following age groups as follows: for 0–2 months 30–80 mg/kg/day, for 3 months–12 years 60–120 mg/kg/day and for 13–18 years 1.5–6 g/day. Dose adjustments were based on clinical indication and an age-appropriate dosing approach [39]. In the case of severe infection such as meningitis, septic shock or shunt infection, a higher dose was recommended to achieve target plasma concentrations of meropenem in the form of 20–40 µg/mL and a duration of treatment of 7–21 days. In 33 of the cases, dose regimens were adjusted according to plasma concentrations lower or higher than the target 20–40 µg/mL, according to the treatment protocol.

### 4.3. Bioanalytical Assays

Blood samples were taken from the arterial line. The minimum sample requirement was 5 microliters of plasma or serum or less if an extremely special situation occurred. Meropenem concentrations were measured using a validated liquid chromatography/tandem mass spectrometry (LC-MS/MS) method [40].

Minimum inhibitory concentrations (MICs) were determined in the Microbiology Laboratory of Karolinska University Hospital in Stockholm. MICs for meropenem were determined with the solid-phase culture diffusion method. If the MIC was not available, the epidemiological cutoff value defined by the European Committee for Antimicrobial Susceptibility Testing (EUCAST) was used for the identified pathogens [41].

Only patients with at least two measured meropenem plasma concentrations during the meropenem treatment were included. The exclusion criterion for the study was refusal of the general informed consent letter.

### 4.4. Population Pharmacokinetic Modelling

The population PK analysis was conducted with NONMEM V7.3.0 (ICON Development Solutions, Ellicott City, MD, USA) and PsN v3.4.2, both operated within Pirana 2.9.0 [42,43]. Data visualization and model diagnostics were carried out using R v4.2.2. The model development consisted of three stages.

(1) Development of structural and statistical model: for the structural model, both one- and two-compartment models were explored to depict the distribution of meropenem. A first-order clearance was assumed for meropenem. Inter-individual variability was examined for each PK parameter, assuming log-normal distribution with estimated variance. Various error models, including proportional, additive and combination, were evaluated for the residual error model.
(2) Covariate model: In the next step, the following covariates were tested:

- Maturation variables: BW and PNA were tested as continuous covariates.
- Disease status: laboratory values, including serum creatinine (μmol/L), estimated creatinine clearance (CrCl, calculated according to Schwartz [44,45]), serum urea (mmol/L), serum albumin (g/L) during the whole course of the disease, serum albumin values at the start of treatment (g/L), total bilirubin (μmol/L), blood pH, C-reactive protein (CRP) (mg/L), aspartate transaminase (IU/L), alanine transaminase (IU/L) and the pediatric index of mortality (PIM3) score, were tested as continuous covariates.
- Concomitant therapy: the use of inotropic drugs, diuretics, along with CRRT use were tested as categorical covariates.
- ECMO: ECMO modalities (veno-venous and veno-arterial), on/off ECMO and change in ECMO circuit were tested as categorical covariates; ECMO speed (revolutions/min), ECMO flow (L/min), duration of ECMO treatment (hours) and time after start and stop of ECMO were tested as continuous covariates.

Multiple time-varying measurements were attainable for all continuous covariates. A stepwise covariate modeling procedure was carried out. Continuous covariates were evaluated in linear and power functions, while categorical covariates were assessed by estimating the parameter value for one category as a proportion of the parameter value for the other category. For model selection, a reduction in the objective function of more than 3.84 points between nested models (*p* < 0.05) was deemed statistically significant, based on a chi-square test distribution. Additional criteria for model selection included satisfactory relative standard errors (RSEs) of the structural model parameter estimates, physiologic plausibility of the obtained parameter values and the absence of bias in goodness-of-fit (GOF) plots.

(3) Validation of the final model: A bootstrap analysis was carried out to determine the stability of the final model. In this process, 1000 replicates of the original data were created by sampling patients from the original dataset with replacements. The final model was applied to each of these 1000 resampled datasets, followed by comparing the median and 95% confidence intervals (CIs) obtained for each parameter with the estimates in the final model. The predictive capabilities of the structural and statistical model were checked using normalized prediction distribution errors (NPDEs). To achieve this, the original dataset was simulated 500 times and the observed concentrations were then compared to the range of simulated values using the NPDE package developed for R (R Foundation for Statistical Computing, Vienna, Austria; http://www.R-project.org, 3 January 2024) [46].

### 4.5. Monte Carlo Simulations

Monte Carlo dosing simulations (10,000) were performed for short (0.5 h) and prolonged infusions (3 h) of 20 mg/kg and 40 mg/kg of meropenem every 8 h (q8h) and a continuous regimen of 60 and 120 mg/kg/day for patients with a BW of 7.88 kg (median), as well as 3.92 kg (25th percentile of IQR) and 11.97 kg (75th percentile), on and off CRRT for 7 days. Each simulation produced steady-state concentration–time profiles for 10,000 subjects using the final estimated population PK parameters. From these profiles, the % fT >  MIC was computed for each subject. Subsequently, a probability of target achievement (PTA) was determined by tallying the subjects who attained 40% or 100% fT > MIC across MIC values ranging from 0.5 to 16 mg/L.

## 5. Conclusions

In conclusion, we found that meropenem PK was not affected by ECMO in neonatal and pediatric settings. CRRT, when used under the stated conditions, was associated with a two-fold increase in Vd, and BW was a significant covariate for both Vd and CL. It seems that current dosing regimens provide a high PTA for MIC ≤ 4 mg/L for 40% fT > MIC, but individual dose adjustments are needed for 100% fT > MIC, as a low PTA was found for this PD parameter.

## Figures and Tables

**Table 1:** Clinical characteristics of the patients included in the analysis.

Parameter (Unit)	Value *
Body weight (kg)	7.88 (3.62–11.97)
Sex, male/female, n (%)	23/22 (51/49)
Age (days) at start of the therapy	3 (0–465)
Neonates, n (%)	27 (60)
Infants (28 days–1 year), n (%)	4 (9)
Children, n (%)	14 (31)
Dosing	
ECMO off	20 to 40 mg/kg 2–3 times a day
ECMO on	loading dose of 20 mg/kg (maximum 2 g) + continuous infusion
Continuous infusion:	
Age group 0–2 months	30–80 mg/kg/day
Age group 3 months–12 years	60–120 mg/kg/day
Age group 13–18 years	1.5–6 g/day.
Laboratory values	
Creatinine (μmol/L)	45 (29–67)
Estimated creatinine clearance (mL/min/1.73 m^2^)	63 (34–113)
Urea (mmol/L)	5.6 (4.2–9)
Total bilirubin (μmol/L)	15 (6–49)
AST (IU/L)	0.96 (0.53–2.18)
ALT (IU/L)	0.66 (0.31–1.33)
pH of blood	7.39 (7.33–7.43)
Albumin (g/L)	26 (22–30)
Albumin at the start of treatment (g/L)	23 (22–26)
CRP (mg/L)	49 (18–112)
PIM3 score	30.5 (13.4–66.1)
Concomitant treatments	
Diuretics; n (%)	38 (84)
Inotropes; n (%)	41 (91)
CRRT; n (%)	31 (69)
ECMO properties	
ECMO patients; n (%)	38 (84)
Length (h)	230 (107–251)
ECMO flow (L/min)	360 (300–680)
ECMO speed (revolutions/min)	2400 (2150–2800)
Veno-venous modality, n (%) of ECMO patients	7 (18.5)
Veno-arterial modality, n (%) of ECMO patients	31 (81.5)

* Values are presented as median (interquartile range) unless stated otherwise. Abbreviations: ALT—alanine transaminase; AST—aspartate transaminase; CRP—C-reactive protein; CRRT—continuous renal replacement therapy; ECMO—extracorporeal membrane oxygenation; PIM3—pediatric index of mortality 3.

**Table 2:** Parameter estimates of the final model.

Parameter [Units]	Final Model (RSE %)	Bootstrap (95% CI)
Fixed effects		
CL [L/h] = CLp × (BW/7.88)		
CLp	1.09 (8%)	1.09 (0.98–1.21)
Vd [L] = Vp × (BW/7.88) × (1 + θCRRT) ^CRRT_on^		
Vp	3.98 (14%)	4.01 (2.22–7.30)
θCRRT	1.04 (76%)	1.06 (0.14–2.20)
Inter-individual variability		
CL (%)	0.0887 (48%)	0.0882 (0.0429–0.1510)
Vd (%)	0.916 (68%)	0.800 (0.060–1.729)
Residual variability		
Proportional	0.17 (17%)	0.167 (0.119–0.225)

Abbreviations: RSE—relative standard error of the estimate; CL—clearance; CLp—population clearance value; Vd—volume of distribution; Vp—population volume of distribution value; CRRT_on—binary parameter indicating whether CRRT treatment was on (1) or off (0); θCRRT—increase in Vd when CRRT treatment was on.

**Table 3:** Probability of target attainment for 40% fT > MIC and 100% fT > MIC.

Probability of target attainment for 40% fT > MIC												
	20 mg/kg 30 min q8h		20 mg/kg 3 h q8h		40 mg/kg 30 min q8h		40 mg/kg 3 h q8h		60 mg/kg/day cont.		120 mg/kg/day cont.	
MIC	CRRT = 0	CRRT = 1	CRRT = 0	CRRT = 1	CRRT = 0	CRRT = 1	CRRT = 0	CRRT = 1	CRRT = 0	CRRT = 1	CRRT = 0	CRRT = 1
0.5	93	98	98	99	95	98	98	99	100	100	100	100
1	91	97	97	99	93	98	98	99	100	100	100	100
2	87	96	95	99	90	98	97	99	100	100	100	100
4	80	93	92	98	87	96	95	99	100	100	100	100
8	55	64	71	78	79	81	82	85	99	99	100	100
16	17	28	35	42	55	65	69	70	45	53	69	69
Probability of target attainment for 100% fT > MIC												
	20 mg/kg 30 min q8h		20 mg/kg 3 h q8h		40 mg/kg 30 min q8h		40 mg/kg 3 h q8h		60 mg/kg/day cont.		120 mg/kg/day cont.	
MIC	CRRT = 0	CRRT = 1	CRRT = 0	CRRT = 1	CRRT = 0	CRRT = 1	CRRT = 0	CRRT = 1	CRRT = 0	CRRT = 1	CRRT = 0	CRRT = 1
0.5	74	81	76	87	75	84	77	89	74	89	79	90
1	71	75	72	83	74	81	76	87	61	84	74	89
2	66	68	61	74	71	75	72	82	55	71	61	74
4	57	51	53	53	65	67	70	74	25	53	54	71
8	12	12	11	13	66	50	52	54	6	21	25	52
16	0	0	0	0	0	0	0	0	0	0	0	0

## Data Availability

The data are not available in any public repository. The model codes are to be made available through the model repository of DDMoRe available through: http://repository.ddmore.foundation/.

## Supplementary Materials

The following supporting information can be downloaded at: https://www.mdpi.com/article/10.3390/antibiotics13050419/s1.
