# Supplementary material for: Meropenem Disposition in Neonatal and Pediatric Extracorporeal Membrane Oxygenation and Continuous Renal Replacement Therapy
Source: Antibiotics (Basel). 2024 May 3;13(5):419. doi: 10.3390/antibiotics13050419 (PMC11117356; doi:10.3390/antibiotics13050419)
Supplement: Supplementary file 1 [file antibiotics-13-00419-s001.zip › antibiotics-2963641-supplementary.pdf]

Figure S1. Normalized prediction distribution errors (NPDE) of the final model. Visual output of the NPDE analysis. Shown at the top are the QQ plot and histogram of the NPDEs in the overall dataset. The red dotted line and blue shaded areas show the expected trends and 95% confidence intervals of these trends, while the dark blue lines and bars show the observed NPDE distributions. At the bottom, the individual NPDE values for each observation are plotted versus time and versus the predicted concentrations with the symbols. The solid lines in the bottom graphs indicate the mean (red) and the 95% percentiles (blue) of the NPDEs, and the shaded areas are the simulated 95% confidence intervals of the NPDE median (red) and 95% percentiles (blue), while the dotted red and blue lines show the expected values for the median and 95% percentiles.

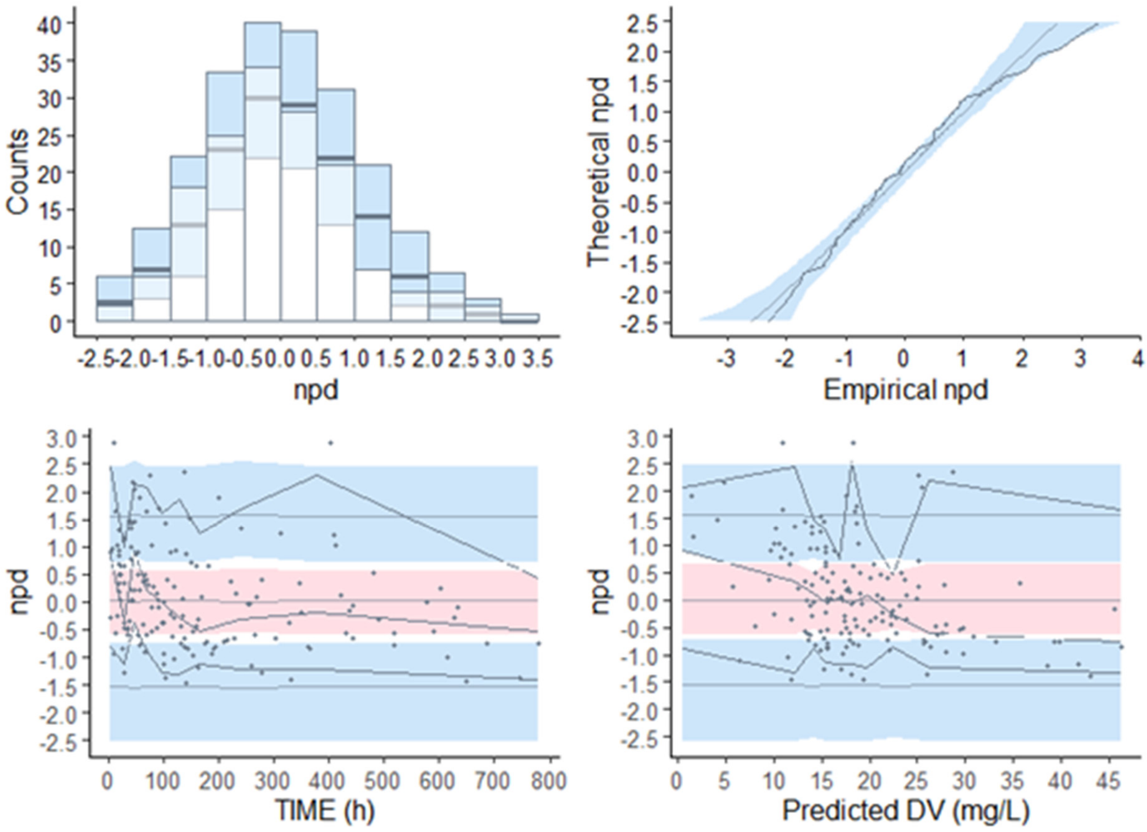

Table S1. Meropenem concentrations distribution on extracorporeal membrane oxygenation (ECMO) and continuous renal replacement therapy (CRRT)

**Modality**	**Number of concentrations (%)**
ECMO on	94 (62%)
CRTT on	91 (60%)
ECMO on, CRRT on	66 (43%)
ECMO off, CRRT on	25 (16%)
ECMO on, CRRT off	28 (18%)
ECMO off, CRRT off	33 (22%)
Pre-ECMO	0
Post-ECMO	37 (24%)

Table S2. PTA for 40%fT>MIC

BW=3.62 kg	20 mg/kg 30 min q8h		20 mg/kg 3 h q8h		40 mg/kg 30 min q8h		40 mg/kg 3 h q8h		60 mg/kg cont.		120 mg/kg cont.	
MIC	CRRT=0	CRRT=1	CRRT=0	CRRT=1	CRRT=0	CRRT=1	CRRT=0	CRRT=1	CRRT=0	CRRT=1	CRRT=0	CRRT=1
0.5	92	98	98	99	94	98	98	99	100	100	100	100
1	90	97	98	99	93	98	97	99	100	100	100	100
2	86	96	95	99	90	97	96	99	100	100	100	100
4	80	93	92	98	86	96	95	99	100	100	100	100
8	65	84	84	85	79	83	82	98	99	100	100	99
16	0	00	0	0	0	0	0	0	0	0	0	0
BW=11.97 kg	20 mg/kg 30 min q8h		20 mg/kg 3 h q8h		40 mg/kg 30 min q8h		40 mg/kg 3 h q8h		60 mg/kg cont.		120 mg/kg cont.	
	CRRT=0	CRRT=1	CRRT=0	CRRT=1	CRRT=0	CRRT=1	CRRT=0	CRRT=1	CRRT=0	CRRT=1	CRRT=0	CRRT=1
0.5	92	98	98	99	94	98	98	99	100	100	100	100
1	90	97	97	99	93	98	97	99	100	100	100	100
2	86	96	95	99	90	97	96	99	100	100	100	100
4	80	93	92	98	86	96	95	99	100	100	100	100
8	65	74	74	79	79	83	79	88	99	99	100	100
16	0	0	0	0	0	0	0	0	0	0	0	0

Table S3. PTA for 100%fT>MIC

BW=3.62 kg	20 mg/kg 30 min q8h		20 mg/kg 3 h q8h		40 mg/kg 30 min q8h		40 mg/kg 3 h q8h		60 mg/kg cont.		120 mg/kg cont.	
MIC	CRRT=0	CRRT=1	CRRT=0	CRRT=1	CRRT=0	CRRT=1	CRRT=0	CRRT=1	CRRT=0	CRRT=1	CRRT=0	CRRT=1
0.5	75	81	73	81	70	84	77	86	85	89	94	98
1	67	74	72	78	74	81	82	89	51	78	79	88
2	47	62	49	48	65	84	72	78	22	49	51	78
4	8	8	8	10	46	61	48	50	4	18	22	45
8	0	0	0	0	7	7	7	10	1	0	5	17
16	0	0	0	0	0	0	0	0	0	0	0	0
BW=11.97 kg	20 mg/kg 30 min q8h		20 mg/kg 3 h q8h		40 mg/kg 30 min q8h		40 mg/kg 3 h q8h		60 mg/kg cont.		120 mg/kg cont.	
	CRRT=0	CRRT=1	CRRT=0	CRRT=1	CRRT=0	CRRT=1	CRRT=0	CRRT=1	CRRT=0	CRRT=1	CRRT=0	CRRT=1
0.5	71	84	77	86	74	85	80	87	85	88	89	90
1	66	81	73	82	81	84	83	86	80	83	85	88
2	58	66	64	71	75	76	77	80	73	79	74	81
4	41	57	43	48	65	67	60	74	25	63	54	68

8	12	12	11	13	50	55	52	54	6	21	25	52
16	0	0	0	0	0	0	0	0	0	0	0	0
